# Irish Medical Schools

**Published:** 1898-09-03

**Authors:** 


### IRISH MEDICAL SCHOOLS.

DUBLIN.

School of Physic in Ireland.

Catholic University Medical School.

The Schools of Surgery of the Royal College of Surgeons in Ireland.

BELFAST.

Queen's College, in connection with the Royal University.

CORK.

Queen's College, in connection with the Royal University.

